# Femoral Neck Osteochondroma Managed With Partial Surgical Excision and Prophylactic Screw Fixation

**DOI:** 10.7759/cureus.116883

**Published:** 2026-09-24

**Authors:** Pavlos Mouratidis, Aristeidis Koutsopoulos, Chrysoula Argyrou, Antiopi Lymperopoulou, Georgios Sarantopoulos, Konstantina Eythymiou, Panagiotis Poalas, Stathis Tsatas, Georgios Kakanis, Vasileios Polyzois

**Affiliations:** 1 4th Department of Orthopaedics, KAT Attica General Hospital, Athens, GRC; 2 Department of Medicine, Laboratory for Research of the Musculoskeletal System “Th. Garofalidis”, National and Kapodistrian University of Athens, Athens, GRC; 3 Department of Medicine, National and Kapodistrian University of Athens, Athens, GRC

**Keywords:** avascular necrosis, benign bone tumor, femoral neck, femoroacetabular impingement, osteochondroma, proximal femur, screw fixation

## Abstract

Osteochondroma is the most common benign bone tumor, characterized by an osteocartilaginous outgrowth with cortical and medullary continuity to the parent bone. While proximal femoral involvement is relatively frequent, particularly in patients with hereditary multiple exostoses, an osteochondroma arising specifically from the femoral neck is uncommon and carries distinct clinical significance due to its intra-articular location. Such lesions can produce femoroacetabular impingement, restricted hip motion, labral and chondral injury, subluxation, acetabular dysplasia, snapping hip phenomena, nerve palsy, and premature osteoarthritis. The proximity of femoral-neck osteochondromas-especially those situated posteriorly or posteroinferiorly-to the deep branch of the medial femoral circumflex artery, the dominant vascular supply to the femoral head, introduces a unique surgical challenge: achieving complete tumor excision while safeguarding femoral-head perfusion to prevent avascular necrosis (AVN).

We report the case of a 23-year-old woman who presented with limited range of motion of the left hip and discomfort during walking and running. Clinical examination revealed restricted terminal external rotation and extension. Radiographic and cross-sectional imaging (computed tomography (CT) and magnetic resonance imaging (MRI)) demonstrated a stalk-like osseous lesion arising from the posteroinferior femoral neck, covered by a cartilaginous cap, with clear cortical continuity to the parent bone-findings consistent with osteochondroma. The joint capsule was thinned and indented by the mass. Given the symptomatic intra-articular location and proximity to the femoral-head blood supply, surgical excision was performed without formal surgical dislocation of the hip, using a posterior approach. The piriformis was released to enhance exposure, and the lesion was resected piecemeal using a saw and osteotomes, with careful preservation of the medial femoral circumflex artery. The deep inferior portion of the lesion could not be fully excised without risking vascular compromise; therefore, a partial resection was performed to restore functional range of motion while preserving femoral-head perfusion. Prophylactic cannulated screw fixation was performed to reinforce the femoral neck and permit immediate postoperative weight-bearing.

## Introduction

Osteochondroma is the most common benign bone lesion and is characterized by an osteocartilaginous projection with continuity of its cortex and medulla with the parent bone. It usually develops in the metaphysis of long bones and grows until skeletal maturity; approximately 90% of patients have a solitary lesion, whereas multiple lesions occur in hereditary multiple exostoses (HME) [1]. Although proximal femoral involvement is common in HME, an osteochondroma arising from the femoral neck is uncommon and clinically important because it is intra-articular and may cause femoroacetabular impingement, restricted hip motion, labral injury, cartilage damage, subluxation, acetabular dysplasia, snapping hip, nerve palsy, and premature osteoarthritis [2-8]. In young patients, these mechanical effects may be particularly consequential because the hip and proximal femur are still developing. The diagnosis is generally suggested by radiographs demonstrating corticomedullary continuity, meaning the lesion maintains direct bony connection to the underlying bone marrow and cortex, a hallmark feature distinguishing osteochondroma from other osseous lesions, and is further defined by computed tomography (CT) and magnetic resonance imaging (MRI), which clarify the lesion’s relationship to the joint, capsule, labrum, cartilage, and surrounding neurovascular structures [9,10]. MRI is also useful for evaluating the cartilage cap and excluding atypical features suggestive of malignant transformation, although malignant change in osteochondroma is uncommon [11,12]. Surgical excision is indicated for symptomatic lesions, progressive deformity, mechanical impingement, neurovascular compromise, or suspicious imaging findings; however, femoral-neck osteochondromas represent a technically demanding surgical problem. Their proximity to the deep branch of the medial femoral circumflex artery, the principal vascular supply to the femoral head, creates a specific risk of vascular injury and subsequent femoral-head avascular necrosis (AVN) [13,14]. This risk is especially relevant for posterior or posteroinferior lesions because the artery courses near the lesser trochanter and posterior aspect of the proximal femur, where it may be displaced, compressed, or injured during exposure and tumor removal [13,14]. Consequently, a case of femoral-neck osteochondroma in a young patient is important not only because of the rarity of the lesion but also because treatment must achieve excision while preserving femoral-head perfusion and avoiding recurrence, femoral-neck fracture, instability, and long-term degenerative change. In this case, given the lesion's proximity to the medial femoral circumflex artery, a partial rather than complete excision was chosen to protect femoral-head blood supply while confirming intraoperatively that the residual disease did not compromise range of motion.

## Case presentation

The patient, a 23-year-old woman, sought consultation due to limitation of the range of motion of the left hip and discomfort during walking and running. Clinical evaluation revealed limitations in both terminal external rotation and extension at the hip joint. Preoperative range of motion was limited, with internal rotation of 45°, external rotation of 20°, and flexion of 120° and extension of 10° measured supine. Radiographic imaging identified a protruding bony growth emerging from the posteroinferior region of the femoral neck (Figure 1). Subsequent cross-sectional imaging-CT (Figure 2) and MRI-characterized the lesion as a stalk-like osseous structure covered by a cartilaginous layer. The joint capsule appeared significantly thinned, with evidence of indentation and breach by the mass. The lesion exhibited clear cortical continuity with the adjacent femoral neck, a hallmark feature supporting its benign osteocartilaginous origin. On fat-suppressed T2-weighted MRI sequences, the cartilaginous cap manifested as a thin band of increased signal intensity, further supporting the diagnosis of osteochondroma. Because of the lesion’s symptomatic intra-articular location and its close relationship to the femoral-head blood supply, surgical excision was planned without surgical dislocation of the hip. The patient was positioned in the lateral decubitus position, and a posterior approach was used. Following dissection, the short external rotators were identified, and the lesion was palpated deep to the rotator tendons. The piriformis muscle was released to enhance surgical exposure, while the remaining external rotators were carefully dissected and retracted, exposing the osteochondroma protruding through the joint capsule. The lesion was approached, with meticulous attention to preserving the adjacent vascular structures. After identification of its base, the osteochondroma was removed piece by piece using a saw and osteotomes. Limb rotation was used to improve exposure. Range of motion was intraoperatively assessed throughout the resection to ensure an unrestricted final result. The inferior portion of the lesion, however, lay deep within the surgical field and would have necessitated either hip dislocation or tenotomy of the external rotator musculature to achieve complete excision-maneuvers that would have risked compromising the vascular supply to the femoral head. Given this trade-off, the decision was made to perform a partial resection, thereby preserving femoral-head perfusion while still restoring functional range of motion. After the excision, the patient was placed on a traction table in a supine position and, under fluoroscopic guidance, underwent prophylactic cannulated screw fixation with 6.5 mm cannulated screws via the same approach. Given the size of the resected lesion, we determined that additional reinforcement of the femoral neck was warranted to permit immediate postoperative weight-bearing (Figure 3). During closure, the piriformis muscle was repaired to its origin using absorbable sutures. The redundant capsule was repaired with absorbable sutures, and the wound was closed in layers.

**Figure 1:**
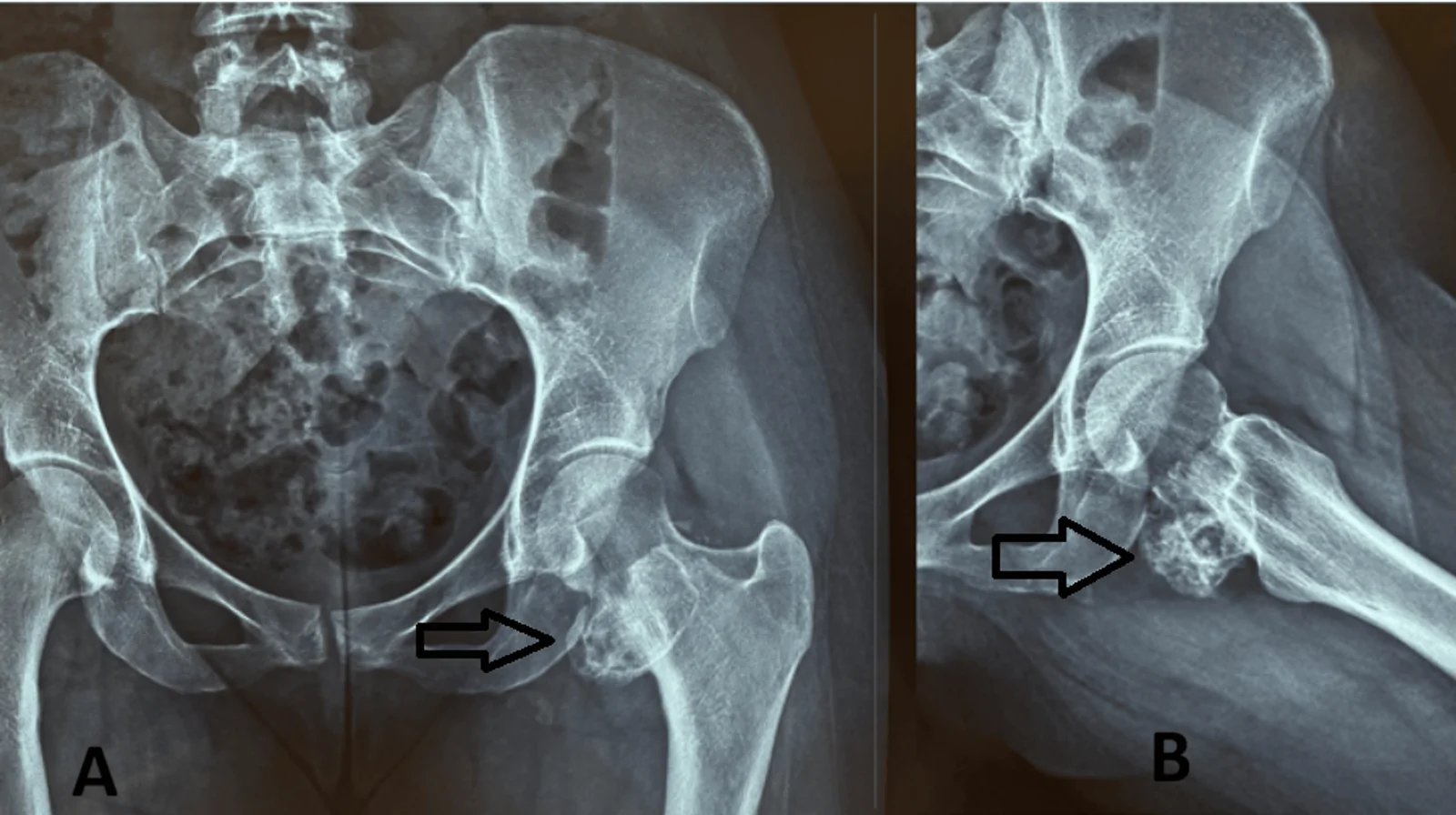
(A) Preoperative pelvis X-ray anteroposterior; (B) preoperative pelvis X-ray lateral Lesion shown at the posteroinferior aspect of the femoral neck

**Figure 2:**
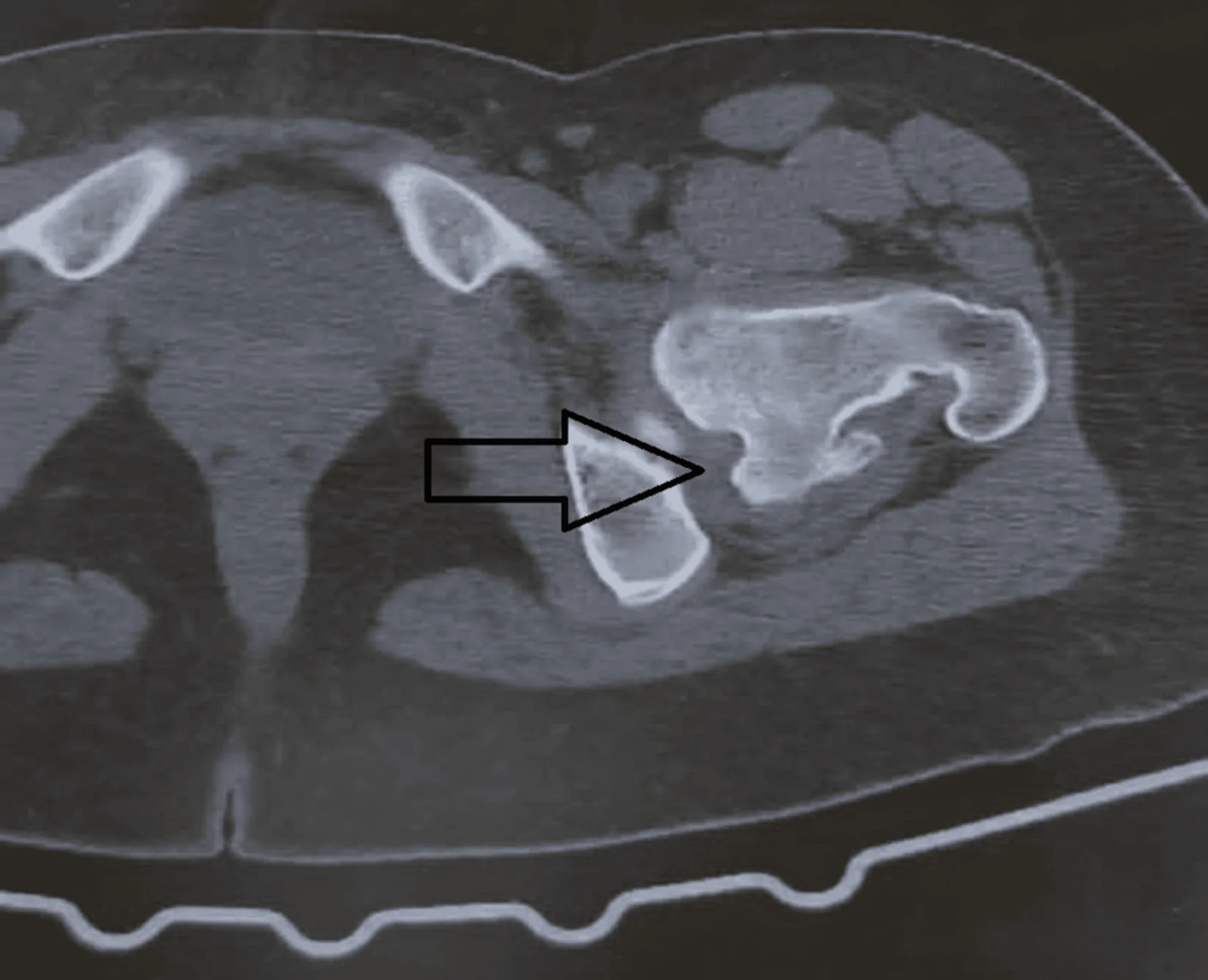
Computed tomography of the left hip Axial section shows the lesion at the posteroinferior part of the femoral neck

**Figure 3:**
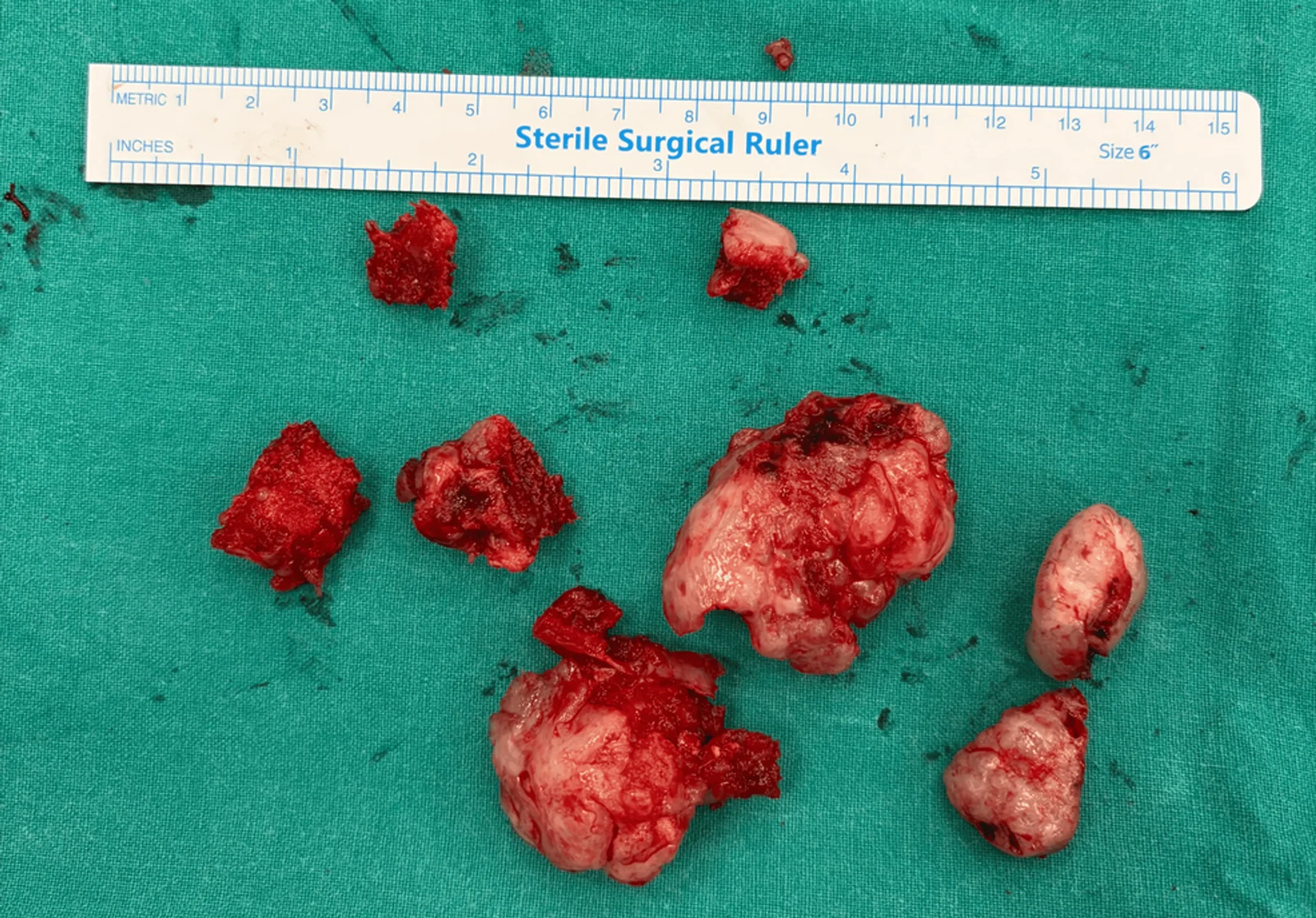
Intraoperative photograph of the resected osteochondroma specimen

Results

The excised specimen was submitted for histopathological examination, which confirmed the diagnosis of osteochondroma. Postoperative range of motion was internal rotation of 45°, external rotation of 60°, and flexion of 120° and extension of 30°. The patient was mobilized immediately postoperatively and permitted full weight-bearing, with complete resolution of preoperative discomfort. At the six-month follow-up, she had returned to normal daily activities, without external rotation restriction. Range of motion remained unchanged from the immediate postoperative period. Radiographic evaluation showed no recurrence. At the six-month follow-up evaluation, both MRI and radiographic imaging showed no evidence of AVN of the femoral head (Figure 4).

**Figure 4:**
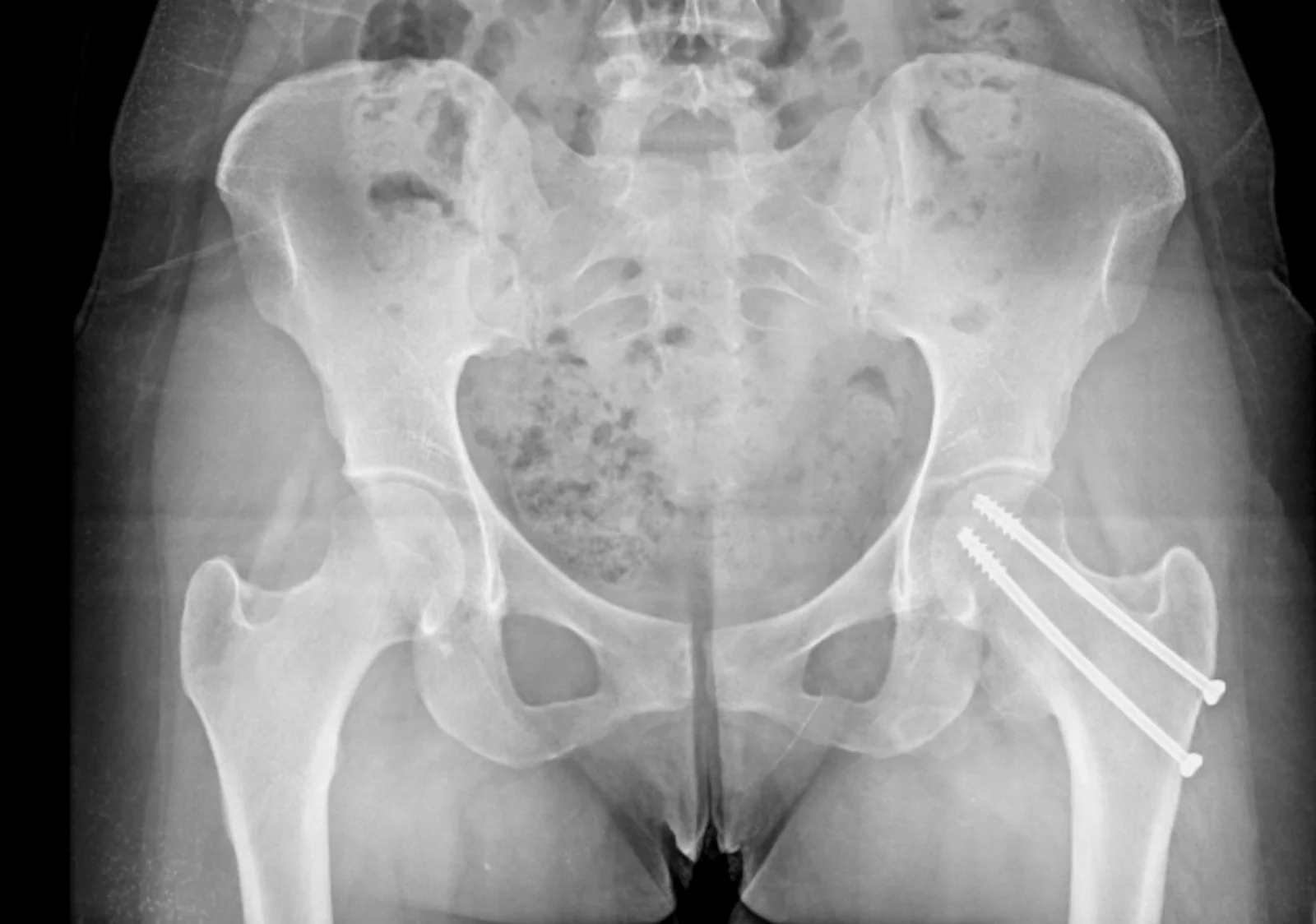
Postoperative X-ray at 6-month follow-up Postoperative pelvis X-ray shows partial resection of the osteochondroma and cannulated screw fixation

## Discussion

The management of femoral-neck osteochondroma should be individualized according to the lesion’s size, morphology, relationship to the capsule and joint, and precise location on the anterior, medial, inferior, posterior, or posteroinferior femoral neck. Preoperative anteroposterior and lateral radiographs should be supplemented by CT, preferably with three-dimensional reconstruction, to define the bony origin and plan the resection, while MRI is valuable for assessing the cartilage cap, labrum, articular cartilage, capsule, and adjacent soft tissues [9,10,15]. For small, predominantly anterior or anteromedial lesions, an anterior approach such as the Smith-Petersen or Hueter approach may provide direct access without hip dislocation; this approach has also been used when associated acetabular dysplasia requires a shelf procedure or periacetabular osteotomy [2]. Nevertheless, anterior exposure may be limited for posterior lesions and has been associated with complications involving the femoral vein and sciatic nerve [13]. Posterior or posteroinferior lesions may be approached through a posterolateral incision with careful identification and protection of the short external rotators and the medial femoral circumflex artery. Mini-arthrotomy without dislocation is an attractive option for selected lesions, particularly when the tumor can be palpated or visualized after separating the external rotators; this technique limits disruption of the femoral-head blood supply and has produced favorable results in young patients [16,17]. For large, circumferential, posterior, inferior, or difficult-to-access lesions, the lateral approach with a trochanteric flip or digastric trochanteric osteotomy and controlled surgical subluxation or dislocation provides the most comprehensive exposure of the femoral neck and permits inspection and treatment of associated labral or chondral pathology [13,14,18]. Siebenrock and Ganz reported complete resection of lesions involving anterior, inferior, and posterior portions of the neck without radiographic or clinical AVN, emphasizing preservation of the obturator externus and the deep branch of the medial femoral circumflex artery [13]. However, surgical dislocation should not be considered risk-free, particularly in young patients or in those with previous operations, because vascular injury, trochanteric complications, femoral-neck fracture, and postoperative osteonecrosis remain possible. During posterior or posterosuperior resection, the artery and its terminal retinacular branches must be directly protected; preoperative angiography or vascular imaging may be considered when a large lesion appears to displace or encase the artery [13,14]. In selected patients with small, accessible, completely intra-articular lesions, hip arthroscopy offers a minimally invasive alternative. Arthroscopic techniques may include central-compartment assessment, labral treatment, traction release, extension into the peripheral compartment, fluoroscopically guided osteochondroplasty, and capsular closure or plication [15,19-22]. Arthroscopy is particularly attractive for anterior or anteromedial lesions, although it remains technically demanding for distal, posterior, or bulky tumors and should be reserved for experienced surgeons. Navigation-assisted excision has also been described as a method of improving localization and controlling the depth of resection in anatomically complex proximal femoral lesions [16]. Regardless of the approach, the objectives are complete removal of the cartilaginous cap, restoration of a nonimpinging femoral-head-neck contour, preservation of sufficient femoral-neck cortex to avoid fracture, and protection of the femoral-head circulation. In managing large lesions, surgeons should recognize that partial resection, rather than complete resection, may represent an optimal strategy when it provides unrestricted hip mobility and protects the femoral-head vascularity. A key limitation of this case report is the relatively short follow-up period of six months, which is insufficient to assess long-term outcomes such as recurrence, progression, or late complications. Another limitation is that the preoperative MRI was lost to follow-up. As a result, no MRI images were available.

## Conclusions

Femoral-neck osteochondroma, though rare, represents a clinically important entity due to its intra-articular location and the mechanical and vascular challenges it poses. Early recognition through targeted radiographic and MRI assessment is essential for accurate diagnosis and surgical planning. This case demonstrates that partial excision without formal surgical dislocation-performed through a posterior approach with careful preservation of the medial femoral circumflex artery-can address symptomatic posteroinferior lesions. Partial resection tailored to restore unrestricted hip motion, combined with prophylactic cannulated screw fixation, can help prevent the major risks associated with this pathology: AVN, fracture, recurrence, and instability. At the six-month follow-up, based on our preliminary, single-case, short-term findings, the patient achieved complete symptomatic resolution, full restoration of hip motion, and no radiographic or MRI evidence of recurrence or AVN.
